# Divergent adaptive immune responses define two types of long COVID

**DOI:** 10.3389/fimmu.2023.1221961

**Published:** 2023-07-20

**Authors:** Jérôme Kervevan, Isabelle Staropoli, Dorsaf Slama, Raphaël Jeger-Madiot, Françoise Donnadieu, Delphine Planas, Marie-Pierre Pietri, Wiem Loghmari-Bouchneb, Motolete Alaba Tanah, Rémy Robinot, Faroudy Boufassa, Michael White, Dominique Salmon-Ceron, Lisa A. Chakrabarti

**Affiliations:** ^1^ Virus and Immunity Unit, Institut Pasteur, Université de Paris Cité, CNRS UMR3569, Paris, France; ^2^ Department of Infectious Diseases and Immunology, Hôtel Dieu Hospital, Assistance Publique-Hôpitaux de Paris, Université de Paris Cité, Paris, France; ^3^ Infectious Disease Analytics and Epidemiology G5 Unit, Institut Pasteur, Université de Paris Cité, Paris, France; ^4^ INSERM U1018, Center for Research in Epidemiology and Population Health (CESP), Le Kremlin-Bicêtre, France

**Keywords:** long COVID, T cell, humoral immune response, seronegative and seropositive, common cold coronavirus, SARS-CoV-2

## Abstract

**Background:**

The role of adaptive immune responses in long COVID remains poorly understood, with contrasting hypotheses suggesting either an insufficient antiviral response or an excessive immune response associated with inflammatory damage. To address this issue, we set to characterize humoral and CD4+ T cell responses in long COVID patients prior to SARS-CoV-2 vaccination.

**Methods:**

Long COVID patients who were seropositive (LC+, n=28) or seronegative (LC-, n=23) by spike ELISA assay were recruited based on (i) an initial SARS-CoV-2 infection documented by PCR or the conjunction of three major signs of COVID-19 and (ii) the persistence or resurgence of at least 3 symptoms for over 3 months. They were compared to COVID patients with resolved symptoms (RE, n=29) and uninfected control individuals (HD, n=29).

**Results:**

The spectrum of persistent symptoms proved similar in both long COVID groups, with a trend for a higher number of symptoms in the seronegative group (median=6 *vs* 4.5; P=0.01). The use a highly sensitive S-flow assay enabled the detection of low levels of SARS-CoV-2 spike-specific IgG in 22.7% of ELISA-seronegative long COVID (LC-) patients. In contrast, spike-specific IgG levels were uniformly high in the LC+ and RE groups. Multiplexed antibody analyses to 30 different viral antigens showed that LC- patients had defective antibody responses to all SARS-CoV-2 proteins tested but had in most cases preserved responses to other viruses. A sensitive primary T cell line assay revealed low but detectable SARS-CoV-2-specific CD4 responses in 39.1% of LC- patients, while response frequencies were high in the LC+ and RE groups. Correlation analyses showed overall strong associations between humoral and cellular responses, with exceptions in the LC- group.

**Conclusions:**

These findings provide evidence for two major types of antiviral immune responses in long COVID. Seropositive patients showed coordinated cellular and humoral responses at least as high as those of recovered patients. In contrast, ELISA-seronegative long COVID patients showed overall low antiviral responses, with detectable specific CD4+ T cells and/or antibodies in close to half of patients (52.2%). These divergent findings in patients sharing a comparable spectrum of persistent symptoms raise the possibility of multiple etiologies in long COVID.

## Introduction

A significant proportion of patients with COVID-19 experience persisting symptoms more than two months after an initial infection with SARS-CoV-2 (1). This post-viral syndrome is termed long COVID, post-acute sequelae of SARS-CoV-2 infection (PASC), or post-COVID-19 condition, as defined by the WHO (2). Long COVID is characterized by a diverse array of symptoms, with a predominance of debilitating fatigue, difficulties in memory and concentration, and dyspnea (3). Additional symptoms include signs of autonomic dysfunction, such as tachycardia and poor regulation of blood pressure, and may also include digestive, renal, reproductive, vascular, and immunological manifestations (4, 5). Early studies suggested a high frequency (above 30%) of persisting symptoms in patients who had been hospitalized for severe COVID-19 (6, 7). It is now clear, however, that patients with an initially moderate or mild form of COVID-19 can also experience prolonged or resurgent symptoms that prevent return to normal life (8, 9). Frequency estimates of long COVID after mild/moderate infection are generally in the 5-20% range, and tend to be higher in women (4, 10, 11). Long COVID also affects the young, with 5 to 10% of infected adolescent reporting persisting symptoms, even though the initial SARS-CoV-2 infection may have been benign and not always associated with seroconversion (12). Preexisting immunity induced by COVID vaccination decreases the risk of long COVID occurrence in some but not all studies (13, 14). Reinfection by SARS-CoV-2 is still associated with a risk of post-acute sequelae, suggesting that the risk of long COVID is not abolished in a highly preinfected population (15). Long COVID symptoms tend to decrease over time, but close to 10% of COVID-19 patients still experience at least one persisting symptom one year after infection (4, 16, 17). Worryingly, long COVID symptoms may persist for at least three years, with debilitating symptoms still present in a subset of patients infected in the initial 2020 wave of the pandemic (18). Considering the high cumulative incidence of SARS-CoV-2 infection worldwide, long COVID is now considered as a significant public health concern (19).

The etiology of long COVID remains poorly understood and is currently the object of a major research effort. One major hypothesis focuses on the persistence of a SARS-CoV-2 reservoir, either in the form of hidden virus replicating at low levels in sanctuary sites, or in the form of non-replicative viral remnants that would chronically stimulates the antiviral response. The punctual detection of viral RNA and/or viral proteins in autopsy material, olfactory mucosa, and gut biopsies months after the acute infection stage supports the possibility of viral persistence (20–24). The presence of viral material may in turn explain moderate but persistent signs of chronic activation in long COVID, including increased levels of circulating inflammatory mediators (25–30), changed patterns of cytokine production by CD4+ T cells (31), and induction of activation and exhaustion markers in CD8+ T cells (32–35). A lack of viral control and ensuing chronic inflammation may point to an intrinsically inefficient antiviral response to SARS-CoV-2, a notion that we aimed to explore in the present study. Intriguingly, long COVID has also been associated with the reactivation of Epstein-Barr virus and other herpesviruses (31, 36, 37), highlighting the possibility of a relatively broad impairment of antiviral responses. Conversely, excessive immune responses with an autoimmune component have also been proposed to play a role in long COVID. It is well documented that acute viral infections are generally followed by a wave of bystander immune activation, which can trigger undesirable immune responses against self-antigens (38). Auto-antibodies to a variety of self-proteins, including nuclear antigens and G-protein coupled surface receptors, have been reported in a subset of long COVID patients (36, 39), but no consistent autoantibody pattern has been associated to long COVID so far (40). Paradoxically, autoantibodies to chemokines were recently reported to be decreased, rather than increased, in long COVID patients compared to patients who recovered from COVID-19 (41), supporting the notion of a moderate but detectable impairment of immune responses in long COVID.

Alternative etiologies proposed for long COVID include persistent tissue damage induced early during the acute stage of infection (4, 42). The variety of organs targeted by SARS-CoV-2 (lungs, heart, gut, brain, kidneys) may help explain the pleiomorphic nature of long COVID symptoms. A possible role for endotheliopathy, leading to disseminated microvascular clots and impaired vascular function, may also account for multiple organ system involvement (43–45). An impairment of cellular metabolism has been reported in the brain of long COVID patients, which may help explain fatigue as well as dysautonomic and cognitive signs of long COVID (21, 46–48). The abnormal activation of mastocytes may also contribute to dysautonomia (30). These proposed mechanisms are not mutually exclusive, as for instance impaired immunity may facilitate viral neuroinvasion and metabolism dysregulation. Unsupervised analyses of electronic heath record data suggest the occurrence of distinct subtypes of long COVID, with predominant cardiac, respiratory, neurological, or digestive symptoms (49). It is thus possible that distinct etiologies underly the diverse array of symptoms in long COVID.

The role of T cell responses in protecting against severe COVID-19 has been clearly established, with an association between the rapid induction of functional SARS-CoV-2-specific T cells in the acute stage and rapid viral clearance (50, 51). Preexisting T cells induced by common cold coronavirus and able to crossreact to SARS-CoV-2 antigens are thought to prevent COVID-19 in certain cases of abortive seronegative infections (52). In contrast, the role of T cell responses in long COVID remains unclear, with reports of exacerbated T cell cytotoxicity and dysregulated cytokine secretion capacity (34) (32, 33), signs of abnormal T cell activation and exhaustion (31, 35, 36), or, in certain cases, presence of weak or undetectable T cell responses (53, 54). Whether altered T cell functions contribute to an immunopathogenic process or to a failure at controlling viral replication remains an open question. To address these issues, we set to further explore the nature of T cell responses in long COVID, using a primary CD4+ T cell line approach that can reveal weak responses that may be missed in *ex vivo* T cell assays.

Long COVID patients were recruited from the observational PERSICOR cohort established at the Hôtel Dieu hospital at the beginning of the pandemic (8). Importantly, patients were recruited before vaccination, which enabled the study of endogenous SARS-CoV-2-specific responses unperturbed by exogenous antigenic stimulation. Early studies had shown that one third of the long COVID patients in the PERSICOR cohort were seronegative by spike ELISA assay, while their spectrum of symptoms was as severe as that of seropositive patients (55, 56). This contrasted with the lower rate of seronegative infection seen in the general population of COVID-19 patients, which ranged from 2 to 24% depending on the study (57, 58). As seronegative patients represented a substantial part of the cohort and had rarely been included in previous long COVID reports, we chose to study this group in parallel to that of seropositive patients. Using highly sensitive antibody and T cell assays, we could document immunological signs of a previous SARS-CoV-2 infection in half of ELISA-seronegative long COVID patients, suggesting the presence of an insufficient antiviral adaptive response in this group. In contrast, seropositive long COVID patients showed persistently high antibody and CD4+ T cell responses, that did not differ in magnitude and breadth from those of individuals who had recovered from COVID-19. These findings provide evidence for divergent antiviral adaptive responses in long COVID, pointing to distinct pathogenic mechanisms underlying symptom persistence.

## Methods

### Patients recruitment

Study participants were recruited in 2021-2022 among patients included in the observational long COVID cohort PERSICOR implemented at the Hôtel Dieu hospital in Paris. Long COVID patients (n=51) were included based on (1) an initial SARS-CoV-2 infection documented by PCR or the conjunction of 3 major symptoms of acute COVID-19 and (2) the persistence or resurgence of at least 3 long COVID symptoms for over 3 months. Blood samples were collected during the chronic phase of long COVID, at least 3 months after the acute stage of infection (median time: 15 months; min-max: 4-26 months). The list of major COVID-19 symptoms and that of long COVID symptoms are reported in **Table 1**. Long COVID patients were subdivided in a seropositive (LC+ group, n=28) and seronegative (LC- group, n=23) based on the results of the SARS-CoV-2 spike-specific ELISA antibody assay performed at the hospital. Of note, SARS-CoV-2 infection was documented by PCR and/or ELISA for all patients in the LC+ group, but for only a subset of patients in the LC- group, as detailed in **Table 2**. Previous SARS-CoV-2 infection was probable in the LC- group based on clinical criteria, but misdiagnosis could not formally be ruled out, and analyses in this group were considered exploratory.

**Table 1:** Clinical characteristics of patients recruited in the PERSICOT study.

	Seronegative long COVID (LC-)*	Seropositive long COVID (LC+)*	Patients with resolved COVID (RE)	p-value LC- vs LC+^#^	p-value LC- vs RE^#^	p-value LC+ vs RE^#^
Enrolled participants: n	23	28	29			
Age in years: median (range)	44.1 (23.0 - 54.2)	48.8 (33.2 - 60.7)	31.00 (19.3 - 64.6)	0.5429	0.1032	**0.0008**
Sex: M / F (%F)	1 / 22 (95.65%)	6 / 22 (78.57%)	12 / 17 (58.62%)	0.4691	**0.0059**	0.236
Time since symptoms onset in months: median (range)	13 (4 – 26)	15 (5 – 19)	6 (3–17)	>0.9999	**<0.0001**	**<0.0001**
SARS-CoV-2 PCR: n positive /n done (% positive)	4 / 20 (20.00%)	13 / 16 (81.25%)	22 / 24 (82.76%)	**0.0004**	**<0.0001**	>0.9999
Acute stage symptoms				p-value LC- vs LC+^#^	p-value LC- vs RE^#^	p-value LC+ vs RE^#^
Anosmia/ageusia: n (%)	9 (39.13%)	19 (67.86%)	13 (44.83%)	0.1272	>0.9999	0.2519
Fever: n (%)	13 (56.52%)	18 (64.29%)	13 (44.83%)	>0.9999	>0.9999	0.4271
Fatigue: n (%)	12 (52.17%)	19 (67.86%)	21 (72.41%)	0.7368	0.3929	>0.9999
Odynophagia: n (%)	10 (43.48%)	7 (25.00%)	3 (10.34%)	0.3955	**0.0194**	0.6129
Myalgia: n (%)	6 (26.09%)	6 (21.43%)	6 (20.69%)	>0.9999	>0.9999	>0.9999
Cough: n (%)	16 (69.57%)	12 (42.86%)	14 (48.28%)	0.1768	0.3876	>0.9999
Dyspnea: n (%)	13 (56.52%)	11 (39.29%)	5 (17.24%)	0.6164	**0.0109**	0.2563
Thoracic pain: n (%)	9 (39.13%)	5 (17.86%)	6 (20.69%)	0.2483	0.3888	>0.9999
Diarrhea: n (%)	8 (34.78%)	3 (10.71%)	9 (31.03%)	0.149	>0.9999	0.2352
Headache: n (%)	10 (43.48%)	13 (46.43%)	17 (58.62%)	>0.9999	0.8433	>0.9999
Number of acute stage symptoms: median (range)	4 (3 – 9)	5 (2 – 7)	4 (0 – 11)	>0.9999	0.7091	0.4706
Long Covid symtoms				p-value LC- vs LC+^##^		
Neurological signs: n (%)	22 (95.75%)	26 (92.86%)		>0.9999		
Cardiothoracic signs: n (%)	22 (95.75%)	25 (89.29%)		0.6173		
Fatigue: n (%)	15 (65.22%)	25 (89.29%)		**0.0477**		
ENT (ear, nose, and throat) signs: n (%)	16 (69.57%°	17 (60.71%)		0.5671		
Cutaneo-mucous and vascular signs: n (%)	17 (73.91%)	12 (42.86%)		**0.0458**		
Digestive signs: n (%)	15 (65.22%)	12 (42.86%)		0.1602		
Myalgia: n (%)	15 (65.22%)	11 (39.29%)		0.0929		
Ophalmological signs: n (%)	9 (39.13%)	5 (17.86%)		0.1202		
Arthralgia / Enthesiopathy: n (%)	8 (34.78%)	8 (28.57%)		0.764		
Fever: n (%)	8 (34.78%)	1 (3.57%)		**0.0071**		
Chills: n (%)	5 (21.74%)	0 (0%)		**0.0143**		
Number of Long Covid symptoms: median (range)	6 (3 – 10)	4.5 (2 – 9)		**0.0109**		
Past medical history				p-value LC- vs LC+^#^	p-value LC- vs RE^#^	p-value LC+ vs RE^#^
Allergy: n (%)	13 (56.52%)	15 (53.57%)	6 (20.69%)	>0.9999	**0.0297**	**0.0378**
Obesity: n (%)	3 (13.04%)	8 (28.57%)	6 (20.69%)	0.5403	>0.9999	>0.9999
Anxiety / depression: n (%)	4 (17.39%)	5 (17.86%)	1 (3.45%)	>0.9999	0.4005	0.3067
Asthma / COPD: n (%)	3 (13.04%)	3 (10.71%)	2 (6.90%)	>0.9999	>0.9999	>0.9999
Chronic heart disease: n (%)	2 (8.70%)	4 (14.29%)	0 (0%)	>0.9999	0.7200	0.1258
Diabetes: n (%)	0 (0%)	1 (3.57%)	0 (0%)	0.769	>0.9999	0.6838
Cancer / Immunosuppression: n (%)	0 (0%)	2 (7.14%)	0 (0%)	0.3186	>0.9999	0.2585

Clinical characteristics are reported for patients in the following groups: seronegative long COVID.

(LC-), seropositive long COVID (LC+), and patients with resolved COVID (RE).

*Seropositivity for SARS-CoV-2 was determined with a Spike-specific ELISA assay.

^#^Kruskal-Wallis test with Dunn’s correction for multiple comparisons.

^##^Mann-Whitney U test. significant P values (<0.05) are reported in bold values.

**Table 2:** Immunological, virological, and clinical criteria available to document SARS-CoV-2 infection in seronegative long COVID patients.

Seronegative long COVID patient ID	# peptides recognized by CD4	% IgG+ cells in S-flow	% IgA+ cells in S-flow	Immunologically probable infection	PCR confirmed infection	Clinically probable SARS-CoV-2 infection	Probable SARS-CoV-2 infection (any criterion)
CL2	0	1.3	0.2	-	yes	-	yes
CL4	1	6.0	0.5	-	-	-	-
CL5	0	63.9	1.3	yes	-	yes	yes
CL6	0	7.5	0.6	-	-	-	-
CL7	2	7.1	0.5	yes	-	yes	yes
CL11	0	36.7	1.4	-	-	-	-
CL12	0	5.1	0.7	-	-	yes	yes
CL13	0	8.2	0.0	-	-	yes	yes
CL15	2	2.4	1.1	yes	-	-	yes
CL16	0	0.9	0.2	-	yes	-	yes
CL17	2	1.0	1.0	yes	-	-	yes
CL21	0	4.1	25.2	yes	-	yes	yes
CL23	1	33.5	1.3	-	-	yes	yes
CL37	3	88.8	65.2	yes	-	-	yes
CL48	0	2.9	1.4	-	yes	-	yes
CL53	0	12.1	2.7	-	-	yes	yes
CL56	4	93.2	7.6	yes	-	-	yes
CL57	2	9.6	2.3	yes	-	-	yes
CL59	0	66.3	1.2	yes	-	yes	yes
CL60	0	0.7	2.0	-	-	-	-
CL65	5	92.6	76.8	yes	yes	yes	yes
CL66	4	ND	ND	yes	-	-	yes
CL68	5	1.8	0.5	yes	-	-	yes
Number of LC- patients: 23	Criterion 1: peptides ≥2	Criterion 2: IgG+>40%	Criterion 3: IgA+>20%	Criteria 1 or 2 or 3	Criterion 4: PCR+	Criterion 5: ≥3 acute symptoms including anosmia/ageusia	Criteria 1 or 2 or 3 or 4 or 5
Number patient fulfilling criterion	9	5	3	12	4	9	19
% patient fulfilling criterion	39.1%	22.7%	13.6%	**52.2%**	17.4%	39.1%	**82.6%**

Criterion 1: a peptide is considered recognized if the CD4+ T cell line IFN-γ or TNF-α response has a stimulation index >2 and is above the limit of sensitivity defined in HD.

Criterion 2: serum concentration of SARS-CoV-2-specific IgG measured in the S-flow assay is >40% labeled spike-HEK cells. ND, not done.

Criterion 3: serum concentration of SARS-CoV-2-specific IgA measured in the S-flow assay is >20% labeled spike-HEK cells.

Criterion 4: positive SARS-CoV-2 PCR assay performed on nasopharyngeal swab or nasal slits.

Criterion 5: At least 3 symptoms including anosmia/ageusia during the acute COVID stage.

Total % of patients with probable SARS-CoV-2 infection are reported in bold values.

The shading in highlights means the patients with immunologically and/or clinically confirmed SARS-CoV-2 infection.

Long COVID patients were compared to convalescent individuals with resolved COVID-19 symptoms (RE group, n=29), who volunteered to participate to the study. Inclusion criteria for the RE group included a previous SARS-CoV-2 infection documented by PCR, a lack of COVID-19 sequelae, a lack of SARS-CoV-2 IgM, and a negative SARS-CoV-2 PCR test within 72h before blood sampling to limit the possibility of reinfection. None of the patients recruited in the study were hospitalized during the acute stage of COVID-19, except for one patient in the LC- group, who did not receive oxygen supplementation. The acute COVID-19 infection was thus considered mild to moderate for all patients studied. The study also included uninfected control individuals (HD group, n=29) who were recruited as volunteer blood donors at the Hôtel Dieu Hospital (n=16; median age 30.0 yrs; min - max: 20.7 - 50.8 yrs; % females: 71.4%) or as anonymous volunteers blood donors at Etablissement Français du Sang during the prepandemic period (n=13). All study participants were recruited prior to receiving a SARS-CoV-2 vaccine, so that immune responses to SARS-CoV-2 could be studied in the absence of antigenic restimulation. Because the study was launched in 2021 while the COVID vaccination campaign was ongoing in France, patients had to be recruited rapidly before they were vaccinated, which precluded a matching for age, sex, and infection duration between the LC groups and the RE group. The PERSICOT (PERsistent Symptoms In COvid - T cell responses) study was promoted by the ESPOIRS association and approved by the Person Protection Committee CPP Sud-Ouest et Outre-Mer 1 under number CPP 1-21-042 ID 12400. All participants gave written informed consent prior to inclusion in the study.

### S-flow assay

IgG and IgA antibodies specific to the SARS-CoV-2 spike were detected by the S-flow assay, which measures antibody binding to spike-expressing HEK 293T cell, as previously described (59). This assay was shown to have a 100% specificity (95% confidence interval [CI], 98.5%–100%) and 99.2% sensitivity (95% CI, 97.69%–99.78%) for COVID-19 patient sera, and to outperform ELISA assays (60).

The spike-expressing cells (293T-S) were generated by transducing HEK 293T cells (ATCC^®^ CRL‐3216™) with a lentivector expressing a codon-optimized Wuhan SARS-CoV- 2 spike protein (GenBank: QHD43416.1). Control 293T cells were transduced with an empty lentivector to assess background staining. The transduced cells were selected with 2.5 ug/mL of puromycin. To perform the S-flow assay, 5 x 10^4 293T-S cells were plated in a 96-well round bottom plate. 50 μL of patient serum diluted 1:300 in MACS buffer (Miltenyi Biotech) was added to the cells, and the mix was incubated for 1 hour at 4°C. The cells were then washed in PBS and stained with a mix of secondary antibodies anti-human IgG-Fc-AF647 (1:600) and anti-human IgA-Alpha-Chain-AF488 (1:200) (Thermo Fisher Scientific) for 30 min at 4°C. Cells were fixed for 10 min in 4% paraformaldehyde and were acquired on an Attune NxT flow cytometer (Life Technologies). Results were analyzed with the FlowJo 10.7.1 software (Becton Dickinson). The background signal was measured in control 293T cells lacking S and subtracted to define the specific signal. The cut-off for antibody positivity was set at 40% IgG+ cells and 20% IgA+ cells, based on previous comparisons in cohorts of SARS-CoV-2 infected and uninfected individuals (60, 61). The mean fluorescence intensity (MFI) of IgG and IgA binding was also reported, as it was found to provide a quantitative measurement of the levels of SARS-CoV-2 specific antibodies (60).

### Luminex antibody assay

A previously described 9-plex bead-based assay (62) was extended to detect antibodies to 30 antigens in 1 μL serum samples. This assay allowed simultaneous detection of antibodies to 30 antigens, including stabilized trimeric Spike ectodomain, receptor binding domain (RBD) of the spike, Nucleocapsid protein (NP), and a Membrane-Envelope fusion protein (ME). The trimeric Spike ectodomains antigens were produced as recombinant proteins for four SARS-CoV-2 variants, namely of the Wuhan ancestral lineage, Alpha, Beta, and Delta variants. We included 8 antigens of 4 seasonal coronaviruses (Spike ectodomain and NP of NL63, 229E, HKU1, OC43). Also included were 6 antigens of other common viruses,which were obtained from Native Antigen (Oxford, United Kingdom): influenza A (HA from the A/Puerto Rico/8/1934 H1N1 strain), measles (nucleoprotein), mumps (nucleoprotein), rubella (virus like particles), adenovirus 5 (hexon protein) and adenovirus 40 (hexon protein).

ME and Spike Sub-unit-2 (S2) SARS-CoV-2 antigens were purchased from Native Antigen (Oxford, United Kingdom) and all other antigens were produced as recombinant proteins at Institut Pasteur. The mass of proteins coupled on beads was optimized to generate a log-linear standard curve with a pool of 27 positive sera prepared from patients with PCR–confirmed SARS-CoV-2 infection. The levels of specific immunoglobulin G (IgG) for each sample were measured in two separate assays.

Each assay was performed in a 96-well, non-binding microtiter plate, where 50 μL of protein-conjugated magnetic beads (250/region/well) and 50 μL of serum diluted 1:100 were mixed and incubated for 30 min at room temperature on a plate shaker. All dilutions were made in phosphate buffered saline containing 1% bovine serum albumin and 0.05% (v/v) Tween-20 (denoted as PBT). Following incubation, the magnetic beads were separated using a magnetic plate separator (Luminex^®^) for 60 seconds and washed thrice with 100 μl PBT. The washed magnetic beads were incubated for 15 minutes with the detector secondary antibody at room temperature on a plate shaker, washed thrice with 100 μl PBT and finally resuspended in 100 μL of PBT. R-Phycoerythrin- (R-PE) conjugated goat or donkey anti-human IgG antibody was used as detector antibody at 1:120 dilution. A positive control pool of serum used in two-fold serial dilutions from 1:50 to 1:102,400 was included on each 96-well plate. Plates were read using a Luminex^®^ MAGPIX^®^ system, which provides a reading of median fluorescence intensity (MFI) for each of the antigens tested.

### Primary CD4+ T cell line response assay

PBMC from patients and healthy donors were depleted of CD8+ cells using CD8 magnetic microbeads (StemCell) and were then plated at 2x10^6^ cells per well in 24-well plates, and cultured in RPMI 1640 medium supplemented with 10% human AB serum, 2 mM L-glutamine, 10 mM HEPES, 100 ug/ml penicillin/streptomycin, and 5 ng/mL recombinant IL-7 (Miltenyi Biotech). To generate SARS-CoV-2-specific primary CD4+ T cell lines, the CD8-depleted PBMC were stimulated with mini-pools of SARS-CoV-2 immunodominant peptides and further grown for 14 days. Each mini-pool consisted in 3 highly purified 20-mers peptides (>99% purity; ProteoGenix) derived from the SARS-CoV-2 N (N104, N221, N328), S (S166, S235, S751) and M (M86, M141, M176) proteins. The sequence of each peptide is reported in **Supplementary Table 1**. These peptides were chosen based on their reported immunodominance in SARS-CoV-2 infected patients and their limited cross-reactivity against common cold coronaviruses (63–66).

For stimulation, each peptide in the minipool was added at a final concentration of 2 µg/mL. Recombinant IL-2 (Proteogenix) was added at a concentration 100 U/ml 2 days after peptide stimulation, and every 2-3 days afterwards. At day 14, cells were restimulated for 6 hours with each individual peptide in the presence of 1 μg/mL Brefeldin-A and 2 µM Monensin (BioLegend). Positive controls were generated by stimulating the cell lines with a mix of superantigens. To measure intracellular cytokine production in specific CD4+ T cells, cells were washed, treated with FcR Blocker, and stained for surface antigens with antibodies CD3 BV510 (UCHT1, 1:200), CD4 PE-CF594 (RPA-T4, 1:200), CD8 BV786 (SK1, 1:200), all from Biolegend, and with the Live-dead Fixable Near-IR viability dye (Thermo Fisher Scientific). Cells were fixed and permeabilized using the CytoFix/Cytoperm kit (BD Biosciences) before staining for intracellular cytokines with antibodies IFN-γ PerCP-Cy5.5 (B27, 1:100) and TNF-α PE-Cy7 (Mab11, 1:100) from BioLegend. Fluorescence was acquired on a Attune NxT flow cytometer, and analyzed with the FlowJo 10.7.1 software. Intracellular cytokine production was evaluated in the live CD3+ CD4+ CD8- lymphocyte gate, and the percentage of cytokine-producing cells was determined after subtracting the percentage of cytokine-positive events in unstimulated control cultures. The limit of sensitivity of the assay (LOS) was defined as 2 x SD, with SD being the standard deviation of the percentage of cytokine+ cells in the control HD group. The stimulation index (SI) was defined as the percentage of cytokine+ cells divided by the mean percentage of cytokine+ cells in the HD group, as described in (66).

### Statistical analyses

All statistical analyses were carried out with the GraphPad Prism v9.5 software. Comparisons between two groups were made with the non-parametric Mann-Whitney U test. Comparisons between three groups or more were made with the non-parametric Kruskal-Wallis tests with Dunn’s correction for multiple comparisons. All statistical tests were two-sided. Correlations between two parameters were analyzed by simple linear regression. Correlation matrices report Spearman’s R coefficients between all pairs of continuous variables. P values lower than 0.05 were considered statistically significant, with symbols as follows: * P<0.05; ** P<0.01; *** P<0.001; **** P<0.0001. The nature of statistical tests used is reported in the figure legends.

## Results

### Similar clinical signs in seronegative and seropositive long COVID patients

Patients included in the seronegative long COVID group (LC-, n=23) and the seropositive long COVID group (LC+, n=28) were predominantly female and had persisting symptoms for a median time of 13 and 15 months, respectively (**Table 1**). Patients in the LC- and LC+ groups did not significantly differ in age nor in time since symptom onset, but were more frequently detected as SARS-CoV-2 PCR-positive at time of diagnosis in the LC+ group (81.25% in LC+ *vs* 20.00% in LC-, P=0.0004). The number of COVID-19 symptoms during acute infection was comparable in both groups, with a median number of symptoms ranging from 4 to 5 (**Table 1**). The spectrum of long COVID symptoms was overall similar in both groups, though the number of persistent symptoms tended to be higher in the seronegative group, with a median of 6 symptoms in LC- *vs*. 4.5 symptoms in LC+ (P=0.01). Considering individual symptoms, the most marked difference was the persistence of fever episodes in about one third of seronegative long COVID patients and in only one seropositive long COVID patient (34.78% *vs* 3.57%, P=0.007). Past medical history did not appear to differ between the LC- and LC+ groups (**Table 1**). Taken together, the analysis of clinical parameters showed that manifestations of long COVID were as durable and as severe in the seronegative than in the seropositive group, supporting the rationale to include seronegative long COVID patients in the study.

The group of recovered patients (RE, n=29) had a more balanced sex ratio and tended to be younger than patients in the two long COVID groups (**Table 1**). Patients in the RE group also had a shorter time since symptom onset (median time = 6 months, P<0.0001 compared to both LC- and LC+), and were all recruited before they received a COVID vaccine. This enabled the study of antibody and T cell responses specific to the SARS-CoV-2 virus, without perturbations associated to vaccine-induced anamnestic responses. The median number of acute COVID-19 symptoms was 4 in the RE group, which did not differ significantly from the numbers observed in long COVID groups. A detailed analysis of symptoms showed a less frequent occurrence of odynophagia and dyspnea in the RE group compared to the LC- group (P=0.0194 and P=0.0109, respectively), pointing to possibly less severe respiratory symptoms in recovered patients. Of note, antecedents of allergies were more frequent in long COVID patients than in recovered patients (56.52% and 53.57% in LC- and LC+, respectively, *vs* 20.69% in RE; P<0.05 for both comparisons), consistent with a high frequency atopy previously reported in a long COVID study (67).

### The S-flow assay reveals low but detectable antibody responses in a subset of seronegative long COVID patients

**Figure 1:**
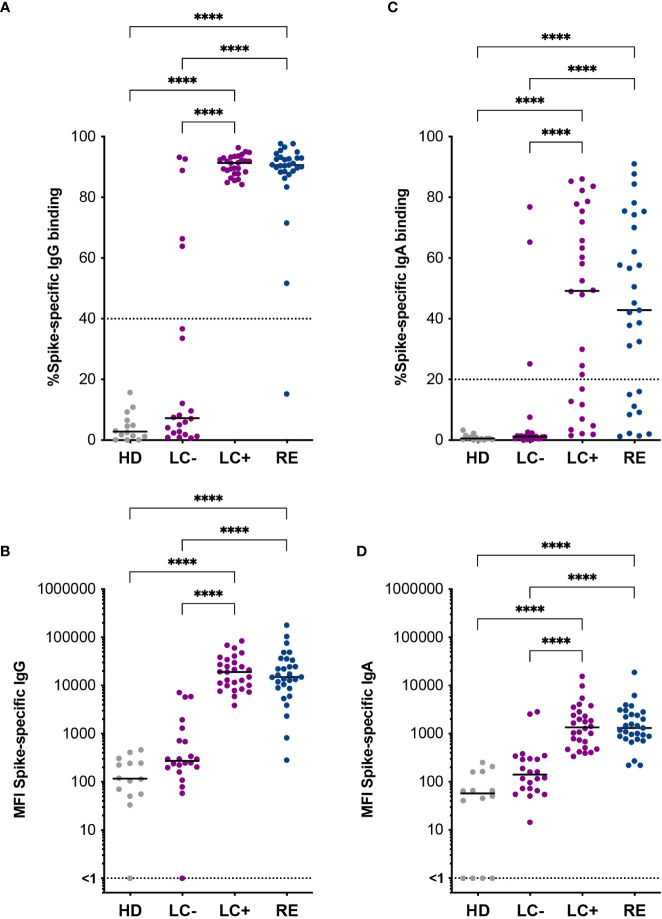
Spike-specific antibodies distinguish two groups of long COVID patients. Antibodies specific to the Wuhan SARS-CoV-2 spike were measured in the S-flow assay. **(A)** Percentage of spike-expressing 293T cells (293T-S) bound by serum IgG. **(B)** Mean fluorescent intensity (MFI) of IgG bound to 293T-S cells. **(C)** Percentage of 293T-S bound by serum IgA. **(D)** MFI of IgA bound to 293T-S cells. HD, healthy donors; LC-, seronegative long COVID patients; LC+, seropositive long COVID patients; RE, recovered patients. Horizontal bars represent medians. Differences between groups were evaluated with the Kruskal-Wallis statistical test with Dunn’s correction for multiple comparisons. ****P<0.0001.

Patients in the LC- group had initially been classified as seronegative based on the SARS-CoV-2 spike ELISA assay used in the clinic. We asked whether a more sensitive antibody detection assay may reveal spike-specific antibody responses in these patients. To this goal, patient sera were analyzed by S-flow, an assay that measures the amount of serum antibodies able to bind HEK 293-T cells stably expressing the Wuhan strain spike protein at their surface (**Figure 1**). This assay has the advantage of measuring antibody binding to the spike in its native conformation and was demonstrated to be more specific and sensitive than classic spike ELISA assays (59, 60). Measuring the frequency of IgG bound spike-expressing 293T cells (IgG+ cells) by flow cytometry showed a negative response in healthy donors (HD) and a strongly positive response in recovered patients, as expected (**Figure 1A**). The frequency of IgG+ cells was as high in the LC+ group as in the RE group, pointing to the persistence of strong spike-specific IgG responses in seropositive long COVID patients. Interestingly, the frequency of IgG+ cells was below the 40% detection threshold for most but not all the seronegative long COVID patients, with 5 LC- patients (22.7%) showing detectable spike-specific IgG, and 2 additional patients having responses just below the detection threshold. These observations were confirmed by an analysis of the mean fluorescence intensity (MFI) of bound IgG, which showed intermediate values in the LC- group, and equivalently high values in the LC+ and RE groups (**Figure 1B**). For consistency, we kept the denomination “seronegative” for all the patients who were included in the LC- group on the basis of a negative ELISA test, even though spike-specific antibodies could be detected by the more sensitive S-flow assay in a subset of these patients.

Spike-specific IgA were also measured by S-flow, as IgA immunoglobulins likely play an important role in controlling SARS-CoV-2 infection in respiratory mucosae. The levels of spike-specific IgA proved more variable and generally lower than that of spike-specific IgG, both in terms of the frequency and the MFI of antibody-bound cells (**Figures 1C, D**). However, a generally similar pattern was observed, with equivalent detection of spike-specific IgA in the LC+ and RE groups, and detection of a few patients (13.6%) with positive IgA responses above the detection threshold in the LC- group. Thus, the use of a sensitive antibody detection assay revealed the presence of detectable spike-specific antibodies in a subset of seronegative long COVID patients, pointing to the possibility of attenuated humoral responses in this group.

### Divergent antibody responses to multiple SARS-CoV-2 proteins in the two groups of long COVID patients

**Figure 2:**
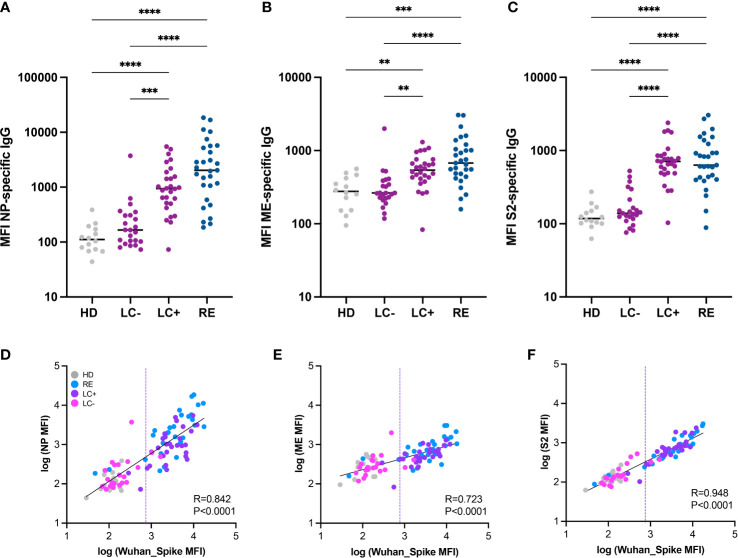
Antibody responses to multiple SARS-CoV-2 proteins distinguish the two groups of long COVID patients. **(A-C)** IgG responses to the nucleoprotein (NP), the M and E proteins (ME), and the S2 spike subunit (S2) from SARS-CoV-2 were measured based on MFI in a multiplexed Luminex assay. Horizontal bars represent medians. Differences between groups were measured with the Kruskal-Wallis test with Dunn’s correction for multiple comparisons. **P<0.01; ***P<0.001; ****P<0.001. **(D-F)** Correlation between antibody responses to different SARS-CoV-2 proteins: NP and Spike **(D)**; ME and Spike **(E)**; and S2 and Spike **(F)**. The linear regression on log-transformed MFI values measured in the Luminex assay is indicated by a straight full line. The linear correlation coefficient R and the P value are reported on each graph. The dashed line represents the threshold for seropositivity estimated by spike antibody measurements in the Luminex assay.

We asked whether the distinction between seropositive and seronegative long COVID patients was valid for antibodies directed to SARS-CoV-2 proteins other than the spike. To this goal, we used a multiplexed Luminex assay developed to measure antibodies to multiple viral proteins simultaneously (62). Analysis of antibodies to the SARS-CoV-2 nucleocapsid (NP) in patient sera showed low responses in the LC- group and strong responses in the LC+ group (**Figure 2A**), indicating that multiple viral proteins induced divergent antibody responses in long COVID. This notion was further confirmed by the detection of low antibody responses to the matrix and envelope (ME) and to the spike S2 subunit in the LC- group, while responses in the LC+ group did not significantly differ from those in recovered patients (**Figures 2B, C**). Further, antibody responses to NP, ME, and S2 showed a strong correlation with antibodies to the Wuhan trimeric Spike measured in the same Luminex assay (**Figures 2D, F**), with correlation coefficients comprised between 0.7 and 0.9 (P<0.0001 in all cases). Again, a few patients from the LC- group showed antibodies to NP, ME, and S2 above those of healthy donors, highlighting the presence of low but detectable antibody responses in a subset of seronegative long COVID patients.

We then verified that the low antibody responses detected in the LC- group were not resulting from a lack of cross-reactivity to antigens of the original Wuhan strain. To this goal, we analyzed responses to spikes derived from the main SARS-CoV-2 VOCs present before and during the patient recruitment period (2020 to early 2022). Antibody measurements to the Wuhan, Alpha, Beta, Delta, Gamma, and Omicron BA.1 spikes showed overall the same patterns in the Luminex assay (**Supplementary Figure S1**). We noted a trend for lower antibody responses directed to the Omicron BA.1 spike in all patient groups, consistent with the notion that most studied patients had been infected prior to the Omicron wave. Taken together, measurements of SARS-CoV-2 specific antibodies consistently showed low or undetectable responses to multiple viral proteins in the LC- group, validating the distinction between the two groups of long COVID patients.

### Similar antibody reactivity to common cold coronaviruses in the two groups of long COVID patients

**Figure 3:**
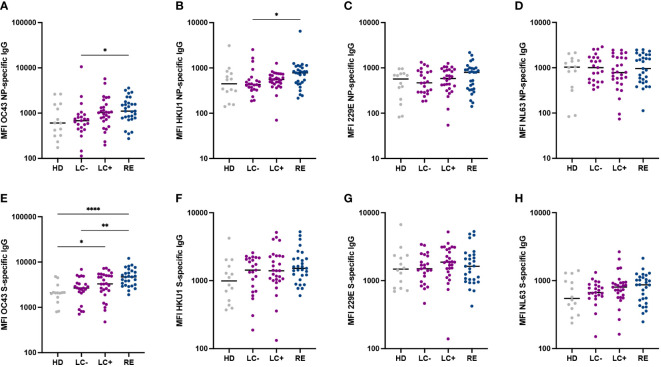
Similar antibody reactivity to common cold coronaviruses in the two long COVID groups. **(A-D)** IgG responses to the nucleoprotein (NP) of the four common cold coronaviruses OC43 **(A)**, HKU1 **(B)**, 229E **(C)** and NL63 **(D)** were measured based on MFI in a multiplexed Luminex assay. **(E-H)** Antibody responses to the spike (S) of the four common cold coronaviruses. Horizontal bars represent medians. Differences between groups were measured with the Kruskal-Wallis test with Dunn’s correction for multiple comparisons. *P<0.05; **P<0.01; **** P<0.0001.

The preexistence of cross-reactive antibody responses to common cold coronaviruses (CCC) has been proposed to play a protective role against the acquisition or the severity of SARS-CoV-2 infection (52). It was therefore of interest to determine whether long COVID patients differed from recovered patients in their humoral responses to CCC. To this goal, we included the spike and NP antigens of the four known human CCC (namely OC43, HKU1, 229E, and NL63) in the Luminex antibody assay (**Figure 3**). Overall, humoral responses to CCC did not show marked differences between the 3 patient groups, suggesting that a strong protective effect of CCC-specific antibodies against long COVID was unlikely. It was however interesting to note that recovered patients had higher antibody responses to OC43 NP (P<0.05), OC43 S (P<0.01), and HKU1 NP (P<0.05) proteins than patients in the LC- group (**Figures 3A, B, E**). These observations may simply reflect the lower SARS-CoV-2 specific antibody responses in the LC- group, resulting in lower cross-reactivity to CCC antigens, but do not rule out the possibility of an intrinsically inefficient humoral response to several human coronaviruses in seronegative long COVID patients.

To examine whether seronegative long COVID patients may mount poor humoral responses to viruses in general, we measured antibody responses to 6 antigens derived from diverse viruses (**Supplementary Figure S2**). Antibody responses to influenza virus A, adenovirus 5 and adenovirus 40 did not differ between the 4 groups, suggesting that long COVID was not associated to a generally lower humoral response to respiratory viruses (**Supplementary Figures S2A–C**). Antibody responses to measles virus and rubella virus antigens did not differ between patient groups but tended to be higher than in healthy controls (**Supplementary Figures S2D, E**), possibly reflecting a degree of persisting bystander activation after SARS-CoV-2 infection. Intriguingly, the LC- group showed lower antibody responses to the mumps nucleoprotein than LC+ and RE groups (P<0.01 and P<0.05, respectively; **Supplementary Figure S2F**).

### Divergent CD4+ T cell responses in the two groups of long COVID patients

**Figure 4:**
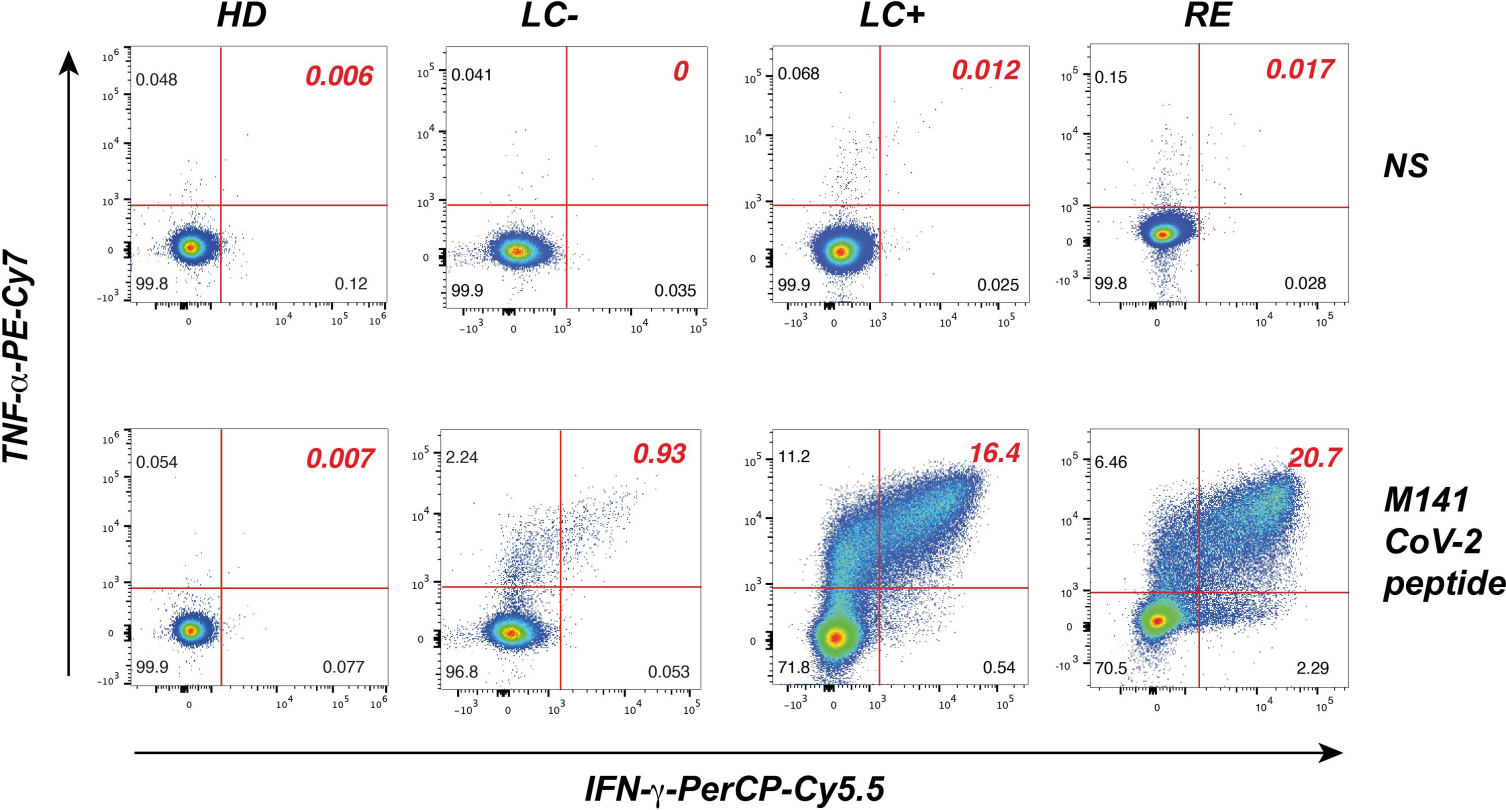
Analysis of SARS-CoV-2 peptide-specific CD4+ T cell responses in primary T cell lines. CD4+ T cell lines restimulated with the matrix M141 peptide (bottom row) or not restimulated (NS, top row), were fixed, permeabilized, and analyzed by flow cytometry in the viable CD3+ CD4+ gate for the intracellular production of IFN-γ (x axis) and TNF-α (y axis). Representative examples of responses are shown for one pre-pandemic healthy donor (HD), one seronegative long COVID patient (LC-), one seropositive long COVID patient (LC+), and one recovered patient (RE).

We next set to evaluate T cell responses to immunodominant epitopes from the SARS-CoV-2 M, N, and S proteins in patient and control groups. We focused on CD4+ T cell responses as they involved in the maturation of the B cell response and may help explain lack of seroconversion. We relied on the generation of primary CD4+ T cells lines, as this approach provides a higher sensitivity of specific T cell detection compared to *ex vivo* T cell analyses. Patient PBMC stimulated by minipools of 3 immunodominant peptides per protein were grown for 14 days, and then restimulated by individual peptides before evaluation by intracellular cytokine staining (ICS). The representative examples provided **Figure 4** shows that after restimulation with the matrix M141 peptide, responses were undetectable in a healthy donor, while a seropositive long COVID patient and a recovered patient showed a strong production of both the IFN-γ and TNF-α cytokines (>15% IFN-γ+ TNF-α+ cells). Interestingly, a weaker but detectable response (0.9% IFN-γ+ TNF-α+ cells) was detected for a seronegative long COVID patient.

**Figure 5:**
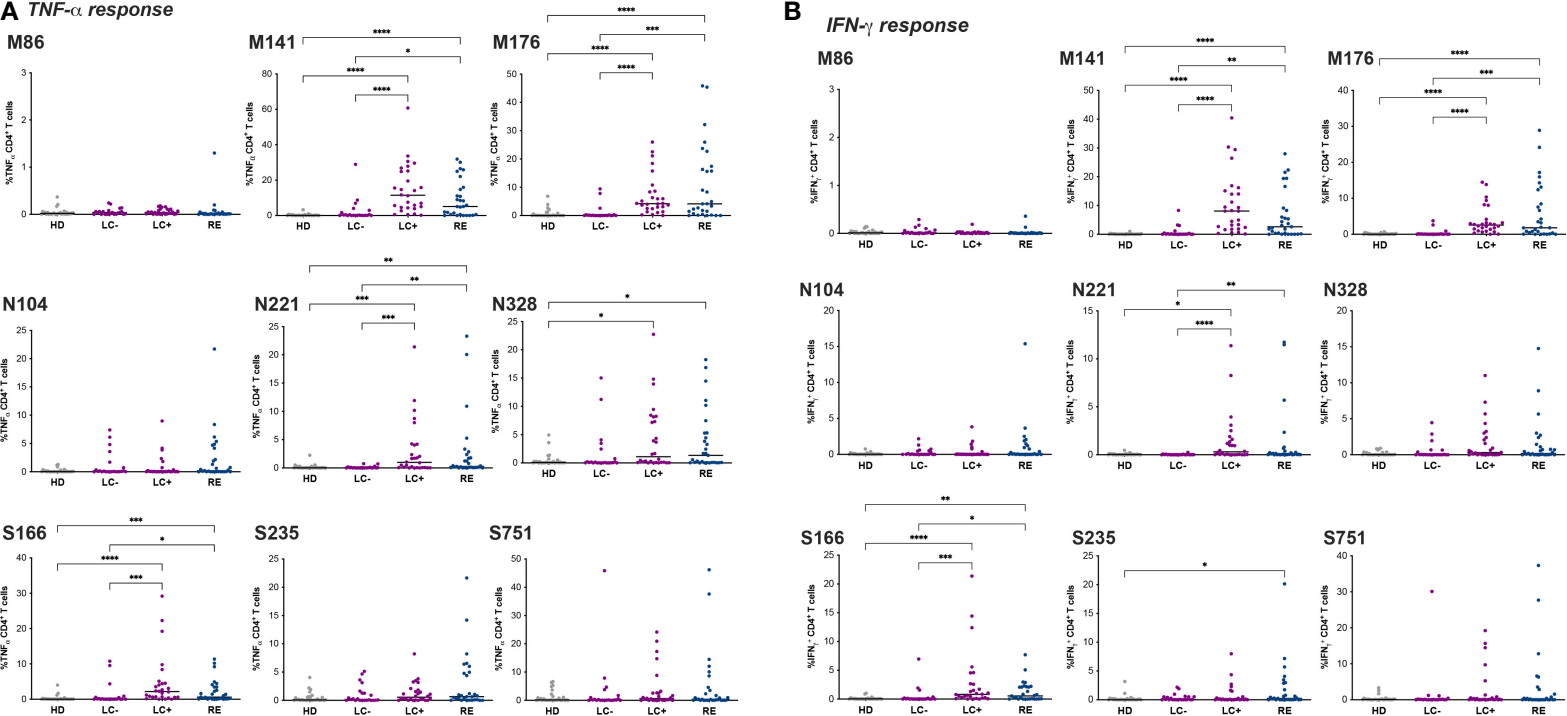
Divergent CD4+ T cell responses in seronegative and seropositive long COVID patients. The frequency of CD4+ T cells producing TNF-α **(A)** and IFN-γ **(B)** in primary T cell lines stimulated with the indicated peptide is reported. Peptides are designated by the viral protein (M: matrix, N: nucleocapsid; S spike) and the position of the first a.a. in that protein. Horizontal bars represent medians. Differences between groups were evaluated with the Kruskal-Wallis statistical test with Dunn’s correction for multiple comparisons. *P<0.05; **P<0.01; ***P<0.001; ****P<0.0001.

Analysis of TNF-α responses (**Figure 5A**) revealed that a majority of patients from the LC+ and RE groups responded to two immunodominant peptides in matrix, M141 and M176, while responses to the third matrix peptide M86 were minimal. Responses to matrix peptides were low in the LC- group, though a few patients (n=4) had responses to M141 above those of heathy donors. Responses to the nucleocapsid and spike peptides were less frequent than those to matrix peptides but showed overall the same pattern, with an equivalent magnitude of responses in the LC+ and RE groups. Again, responses to the nucleocapsid and spike peptides were low in the LC- group, but with responses above those of healthy donors in a subset of patients.

Of note, positive ICS responses were also detected for some individuals in the HD group, likely due to cross-reactivity of CCC-specific CD4+ T cells with SARS-CoV-2 derived peptides (68). However, the strategy of screening CD4+ T cell lines against individual peptides rather than a peptide pool enabled the identification of peptides with a low degree of cross-reactivity in the HD group, such as M141 and N104, supporting the notion that a subset of LC- patients had SARS-CoV-2-specific rather than cross-reactive CD4+ T cell responses. Analyses of IFN-γ production in CD4+ T cell lines showed low levels of responses in the HD group (**Figure 5B**), thus confirming the presence of SARS-CoV-2-specific CD4+ T cells in a few LC- patients. Overall, IFN-γ responses were of lower magnitude than TNF-α responses but followed the same pattern. A trend for higher IFN-γ response to M141 was noted in the LC+ group compared to the RE group, though it did not reach statistical significance.

**Figure 6:**
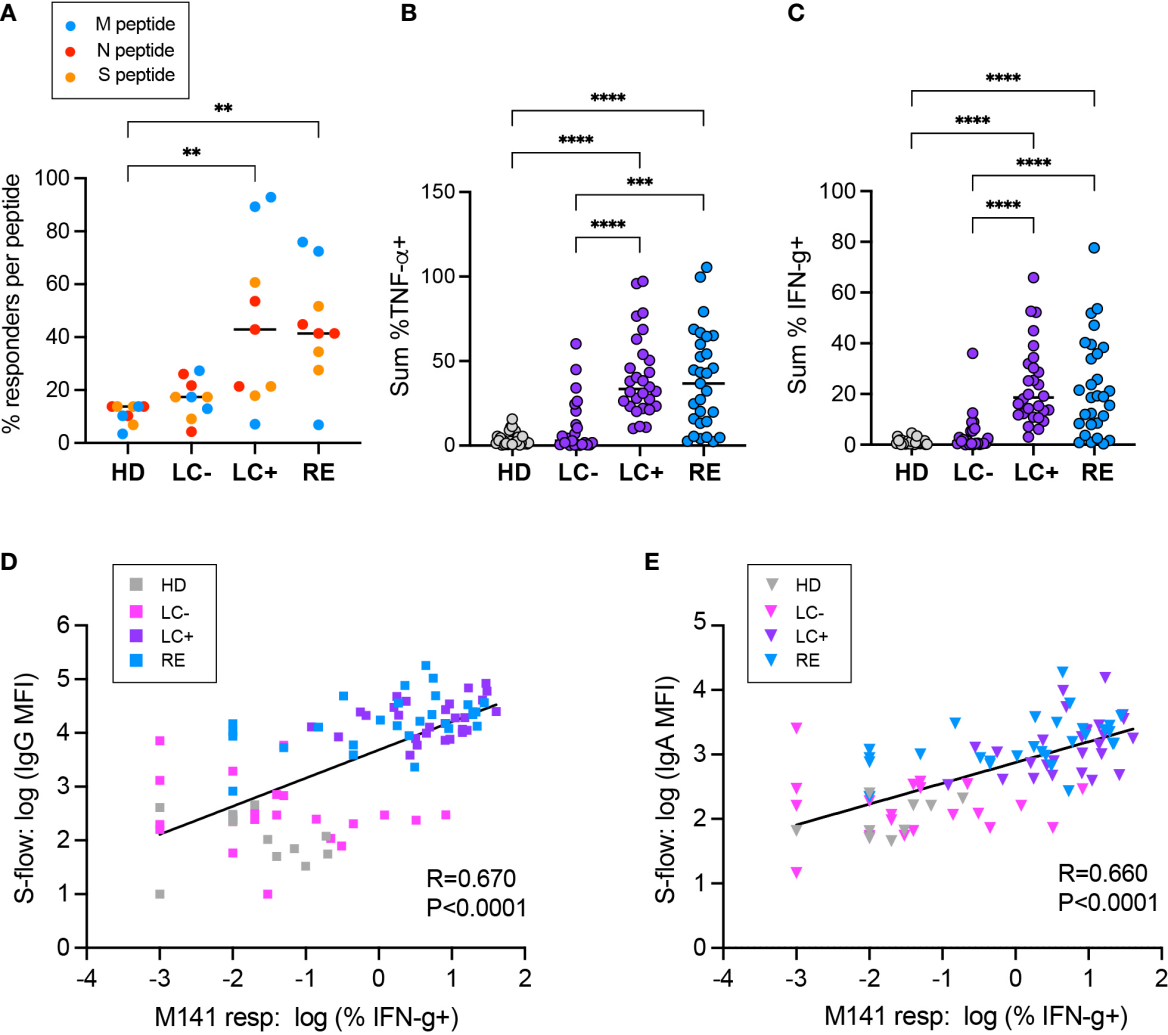
Positive correlation between the CD4+ T cell response and the antibody responses to SARS-CoV-2. **(A)** The percentage of individuals with a CD4+ T cell response to each of the 9 immunodominant peptides tested is reported. A responder is an individual for whom the % TNF-α+ or % IFN-γ+ cells in CD4+ T cells is above the limit of sensitivity (LOS = 2 x SD for the HD group) and has a stimulation index SI >2 for the considered peptide. Peptides derived from the matrix (M), nucleoprotein (N) and spike (S) are color-coded in blue, red, and orange, respectively. **(B, C)** The summed frequency of CD4+ T cells producing TNF-α **(A)** and IFN-γ **(B)** in response to the 9 peptides tested is reported. **(A-C)** Horizontal bars represent medians. Differences between groups were evaluated with the Kruskal-Wallis statistical test with Dunn’s correction for multiple comparisons. **P<0.01; ***P<0.001; ****P<0.0001. **(D, E)** Linear regression between the CD4+ T cell response to the M141 peptide, measured by the % of IFN-γ+ cells, and the antibody response, measured in the S-flow assay by the MFI of spike-cells bound by serum IgG **(D)** or IgA **(E)**. The linear regression coefficient R and the P value for the slope of the regression line being different from zero are reported.

We then computed the frequency of individuals with a detectable CD4+ T cell response to each of the immunodominant peptide tested (**Figure 6A**). A responder was defined as an individual for whom the percentage of IFN-γ+ or TNF-α+ CD4+ T cells was above the limit of sensitivity (LOS) of the assay and had a stimulation index (SI) >2 for the considered peptide (66). Overall, the frequency of responders was higher in the LC+ and RE group than in the HD group (P<0.01 in both cases), while the LC- group showed intermediate response frequencies that were not significantly different from those in other groups. Responses to the matrix M141 peptide appeared the most discriminant between groups, with 92.9% responders in LC+, 72.4% in RE, 27.3% in LC-, and 3.5% in HD. Analyzing the sum of TNF-α or IFN-γ responses for the 9 peptides studied (**Figures 6B, C**) showed a similar hierarchy, with strong responses in the LC- and RE groups, intermediate or low responses in the LC- group, and low responses in the HD group. Taken together, these analyses provided evidence for divergent CD4+ T cell responses in the two groups of long COVID patients, with high magnitude responses in seropositive patients, and low but at times detectable responses in seronegative patients.

### Positive association between CD4+ T cell responses and antibody responses to SARS-CoV-2

To evaluate associations between antiviral cellular and humoral responses, we chose in first intent to compare IFN-γ responses to the M141 peptide and S-flow IgG MFI readings, as these were the parameters that provided the best discrimination between groups (**Figure 6D**). Comparison between these two parameters revealed a positive correlation (R=0.670, P<0.0001), indicating a coordination of the two arms of the antiviral response directed at SARS-CoV-2. We noted however the presence of a few patients of the LC- group with low spike-specific IgG but clearly positive M141 CD4 responses, raising the possibility of a disjunction of cellular and humoral responses in seronegative long COVID. Analysis of the association between IFN-γ responses to M141 and spike-specific IgA supported these observations (**Figure 6E**).

We then visualized the matrix of correlation coefficients between all the clinical and immunological parameters analyzed in the study, considering all the patients and controls included in the study as a single population (**Supplementary Figure S3**). The most apparent feature was the strong correlation between SARS-CoV-2 specific antibodies and SARS-CoV-2-specific CD4 responses. The Spearman correlation coefficients were strongest between antibodies responses and CD4 responses to the two immunodominant matrix peptides or to the summed cytokine responses per viral protein. This analysis also highlighted a negative association between the frequency of long COVID symptoms and SARS-CoV-2 specific responses (humoral and cellular), possibly explained by the presence of low antiviral responses in the seronegative long COVID group. We also noted positive associations between antibody levels to the different CCC proteins. The strongest correlation was observed between antibodies to the NL63 and 229E nucleocapsids, which may be explained by the genetic relatedness of these two alphacoronaviruses, leading to a high frequency of cross-reactive antibodies. The more modest but detectable associations between antibodies to SARS-CoV-2 and to CCC may reflect a moderate degree of antibody cross-reactivity. Similarly, the moderate association between SARS-CoV-2 specific CD4 responses and antibodies of CCC proteins (in particular to the OC43 spike) may reflect antigenic cross-reactivity, though individual variability in the intrinsic capacity to mount responses to human coronaviruses is not ruled out. Last, we noted an unexplained positive association between humoral and cellular responses to SARS-CoV-2 and antibody responses to measles and mumps. Taken together, the global correlation analyses (**Supplementary Figure S3**) confirmed the positive association between the cellular and humoral arms of the antiviral response directed at SARS-CoV-2. However, detailed examination of immune responses within groups (**Figure 6B**) showed instances of poorly correlated cellular and humoral responses among seronegative long COVID patients.

### Immunological signs of SARS-CoV-2 infection are present in half of seronegative long COVID patients

A detailed analysis of immunological findings was carried out in the LC- group, to assess whether the T cell and antibody assays implemented in the study could help document a previous SARS-CoV-2 infection in seronegative long COVID patients (**Table 2**). Patients were considered to have a detectable CD4+ T cell response if they responded to at least 2 SARS-CoV-2 peptides, using the response criteria defined in **Figure 6A** (% of cytokine+ cells >LOS and SI >2). Patients were considered to have a spike-specific IgG response if their serum gave a reading above threshold in the S-flow assay (%IgG+ cells >40%), taking into consideration that the 40% threshold is conservative and has been validated in large cohorts of SARS-CoV-2 infected patients (59, 60). Similarly, patients were considered to have a spike-specific IgA response if their serum resulted in a %IgA+ cells >20% (61). Based on these criteria, 39.1% patients in the LC- group had a detectable CD4 response, 22.7% had spike-specific IgG, and 13.6% had spike-specific IgA (**Table 2**, last line). There was not a complete overlap between the subsets of LC- patients having detectable CD4 and antibody responses, pointing to a lower degree of coordination between cellular and humoral responses in this group. Considering all the three response criteria (CD4, IgG, and IgA), the analysis showed that 52.2% of patients in the LC- group had an immunological sign of a previous SARS-CoV-2 infection.

We next included diagnostic and clinical criteria generally used to document SARS-CoV-2 infection. A limited subset of LC- patients (17.4%) had an infection documented by PCR. In addition, 39.1% of LC- patients fulfilled the definition of having a clinically probable SARS-CoV-2 infection, based on the occurrence of at least 3 listed symptoms including anosmia/ageusia during the acute COVID-19 stage (69). Combining all 5 criteria (CD4, IgG, IgA, PCR, and clinical) showed that 82.6% of patients in the LC- group had signs of a probable SARS-CoV-2 infection (**Table 2**, right column). Taken together, this analysis showed that a majority of seronegative long COVID patients were likely to have been infected by SARS-CoV-2, highlighting the relevance of studying virally induced pathogenic mechanisms in this group.

## Discussion

This study provides evidence for two major types of antiviral immune responses in long COVID patients. The seropositive group showed well coordinated cellular and humoral responses directed at SARS-CoV-2, with levels of specific CD4+ T cells and antibodies that were at least as high as those of recovered patients. In contrast, the group of seronegative long COVID patients showed overall low antiviral responses, with detectable specific CD4+ T cells and/or antibodies in only half of patients. These divergent findings in patients sharing a comparable spectrum of persistent symptoms raise the possibility of multiple etiologies in Long COVID.

The use of highly sensitive immunological assays, namely the S-flow antibody assay and the primary CD4+ T cell line assay, were instrumental in detecting the moderate adaptive responses present in the group of seronegative patients. Combining this immunological evaluation with PCR testing and clinical evaluation helped document a probable SARS-CoV-2 infection in over 80% of these patients. These findings support the idea that a majority of seronegative long COVID patients had previously been infected by SARS-CoV-2, though we cannot entirely rule out other causes for persisting symptoms, such as somatic causes or a post-infectious syndrome due to an unrelated pathogen. Interestingly, a recent study based on a fluorospot assay with enhanced sensitivity reported the presence of detectable T cell responses in close to half of seronegative long COVID patients (54), which appears compatible with our findings, and supports the idea that a subset of long COVID patients have attenuated but detectable responses to SARS-CoV-2. Such attenuated antiviral responses may be insufficient to clear the virus and may promote a degree of viral persistence, which could in turn underly continuous tissue damage and prolonged symptoms. Whether seronegative long COVID patients are more prone to viral persistence remains to be established. In this respect, it was interesting to note that the only notable difference in symptoms between the two long COVID groups was the more frequent occurrence of fever episodes in the seronegative group (34.78% in LC- *vs* 3.57% in LC+). Fever may be a sign of inflammation induced by residual viral replication, viral antigen persistence, or viral reactivation, a notion that deserves further investigation.

It is intriguing to note that a frequent reactivation of herpes viruses has been reported in long COVID (31, 36, 37). These reports raise the possibility that long COVID patients may have inefficient adaptive responses to a variety of viruses, and not only to SARS-CoV-2. In this respect, we noted that patients in the LC- group had lower antibody levels than recovered patients to several CCC proteins, including OC43 NP, HKU1 NP, and OC43S. These observations may point to generally inefficient responses to human coronaviruses, though they may also reflect the lower levels of SARS-CoV-2 specific antibodies in LC- patients, resulting fewer cross-reactive responses to CCC proteins. An evaluation of antibodies to specific CCC epitopes that would not be shared with SARS-CoV-2 may help clarify this issue. Antibody titers to a variety of other viral antigens derived from adenoviruses or influenza, measles and rubella viruses did not differ between the three patient groups. However, antibodies to the mumps viral antigen were significantly lower in seronegative long COVID patients than in the two other groups. These intriguing observations raise the possibility of selectively deficient responses to particular viruses in seronegative long COVID. Of note, a high propensity to allergy was noted in long COVID patients, in the present study (antecedents of allergy in 50% or more of patients in the LC- and LC+ groups, compared to 20% in the RE group) as well as in a previous report (67). A predisposition to allergy may reflect an intrinsic bias in immune responses, which may antagonize the development of interferon-dependent antiviral responses (70), and may thus contribute to long COVID development. Studies of genetic polymorphisms known to influence antiviral immune responses may thus shed light on the mechanisms underlying long COVID.

Antibody and CD4+ T cell responses to SARS-CoV-2 appeared overall well coordinated, with high correlation coefficients between the multiple humoral and cellular parameters measured. However, detailed examination of these parameters in seronegative long COVID patients (**Table 2**) showed a lower degree of coordination in this group, with a frequent disconnect between the detection of IgG, IgA, and CD4+ T cells specific to SARS-CoV-2. One reason may be the low magnitude of these responses, which may be close to the detection threshold of the assays used. However, a few seronegative patients had clearly positive CD4+ T cell responses in the absence of detectable antibodies, raising the possibility that available specific CD4+ T cell had inefficient B cell helper function, and hence could not trigger the maturation of specific antibodies. It may be relevant that a low degree of coordination between cellular and humoral immune responses has been associated with an increased disease severity in acute COVID-19 (51). Further, a longitudinal study showed that lower levels of SARS-CoV-2 specific IgG at 1 to 2 months after COVID-19 onset was associated with a higher rate of persisting symptoms the 4 and 7 months time points (71). Thus, the limited development of the antiviral antibody response may have contributed to the establishment of long COVID in the subset of seronegative patients.

A distinct pathogenic mechanism may be at work in seropositive long COVID patients. We did not detect major differences in the magnitude and breadth of humoral and cellular responses when comparing seropositive long COVID patients to recovered patients. We however noted a trend for a higher frequency of responder patients to the two immunodominant matrix peptides in the LC+ group. Considering that the duration of infection was on average longer in the LC+ group than in the RE group, and that SARS-CoV-2 specific T cell responses are known to progressively decline over time (72), these observations may suggest the presence of relatively strong or persistent T cell responses in seropositive long COVID patients. This would be compatible with previous reports of persistently high T cell responses in Long COVID (34) (32, 33), accompanied in some studies with signs of abnormal T cell activation and T cell exhaustion (31, 35, 36). A limitation of the present study is that we did not evaluate the exhaustion status of specific effector T cells, as we focused on amplifying specific T cells in culture to maximize detection sensitivity. Thus, we do not rule out the possibility of a lower efficiency of effector T cells in long COVID. The presence of numerous but inefficient effector T cells may fail to entirely clear residual infected cells or viral antigen depots, leading to deleterious effects associated to chronic immune activation and persistent inflammation. One may note that chronic immune activation may not only be driven by residual SARS-CoV-2 antigens. A recent study has shown that even mild uncomplicated COVID-19 can impact the basal activation status of the immune system up to five months after the onset of SARS-CoV-2 infection through non-specific bystander mechanisms (73). The phenomenon of bystander activation may for instance explain the increased levels of measles and rubella virus-specific IgG detected in all three patient groups as compared to uninfected controls. It is conceivable that bystander activation may be particularly strong in seropositive long COVID patients, leading to persistent inflammation and/or autoimmunity. This notion is supported by reports of persistently elevated levels of inflammatory markers such as IL-6 in long COVID, suggestive of inflammatory imprinting (25, 26, 35).

Taken together, this study provides evidence for divergent immune responses in long COVID, with one group characterized by low and poorly coordinated humoral and cellular responses, and a second group characterized by strong and persistent responses. These findings raise the possibility of multiple etiologies in long COVID, with persisting symptoms enabled by a deficient antiviral response in seronegative patients, and by immunopathogenic mechanisms that remain to be defined in seropositive patients. The study also highlights the interest of implementing highly sensitive immunological assays to document signs of a previous SARS-CoV-2 infection in seronegative individuals. Seronegative long COVID patients have rarely been included in clinical trials and physiopathological studies thus far, due to the difficulty in documenting their infection by SARS-CoV-2. This may have limited our understanding of long COVID pathogenesis, by excluding a population that represents up to one third of patients. Further, patients without a laboratory documented infection often encounter difficulties in having their condition recognized and treated. This represents a public health concern, as the clinical severity of seronegative long COVID appears as high if not higher as that of the seropositive form, with a higher number of persisting symptoms recorded in the present study. Thus, further development and validation of highly sensitive immunological assays for SARS-CoV-2 is warranted, to help document infection in seronegative long COVID patients and facilitate their access to medical care.

## Data availability statement

The raw data supporting the conclusions of this article will be made available by the authors, without undue reservation.

## Ethics statement

The studies involving human participants were reviewed and approved by the Person Protection Committee CPP Sud-Ouest et Outre-Mer 1 and approved under number CPP 1-21-042 ID 12400. The patients/participants provided their written informed consent to participate in this study.

## Author contributions

Protocol design and clinical management: DS-C, FB, DS, M-PP, WLB, MAT; Experimental design and procedures: JK, IS, RJ-M, FD, DP, RR, LC; Visualization: JK, MW, LC; Supervision and funding acquisition: LC, DS-C, MW; Writing – original draft: LC; Writing – review & editing: DS-C, JK, MW, IS; All authors contributed to the article and approved the submitted version.
