# Supplementary material for: Divergent adaptive immune responses define two types of long COVID
Source: Front Immunol. 2023 Jul 20;14:1221961. doi: 10.3389/fimmu.2023.1221961 (PMC10408302; doi:10.3389/fimmu.2023.1221961)
Supplement: Supplementary file 1 [file DataSheet_1.pdf]

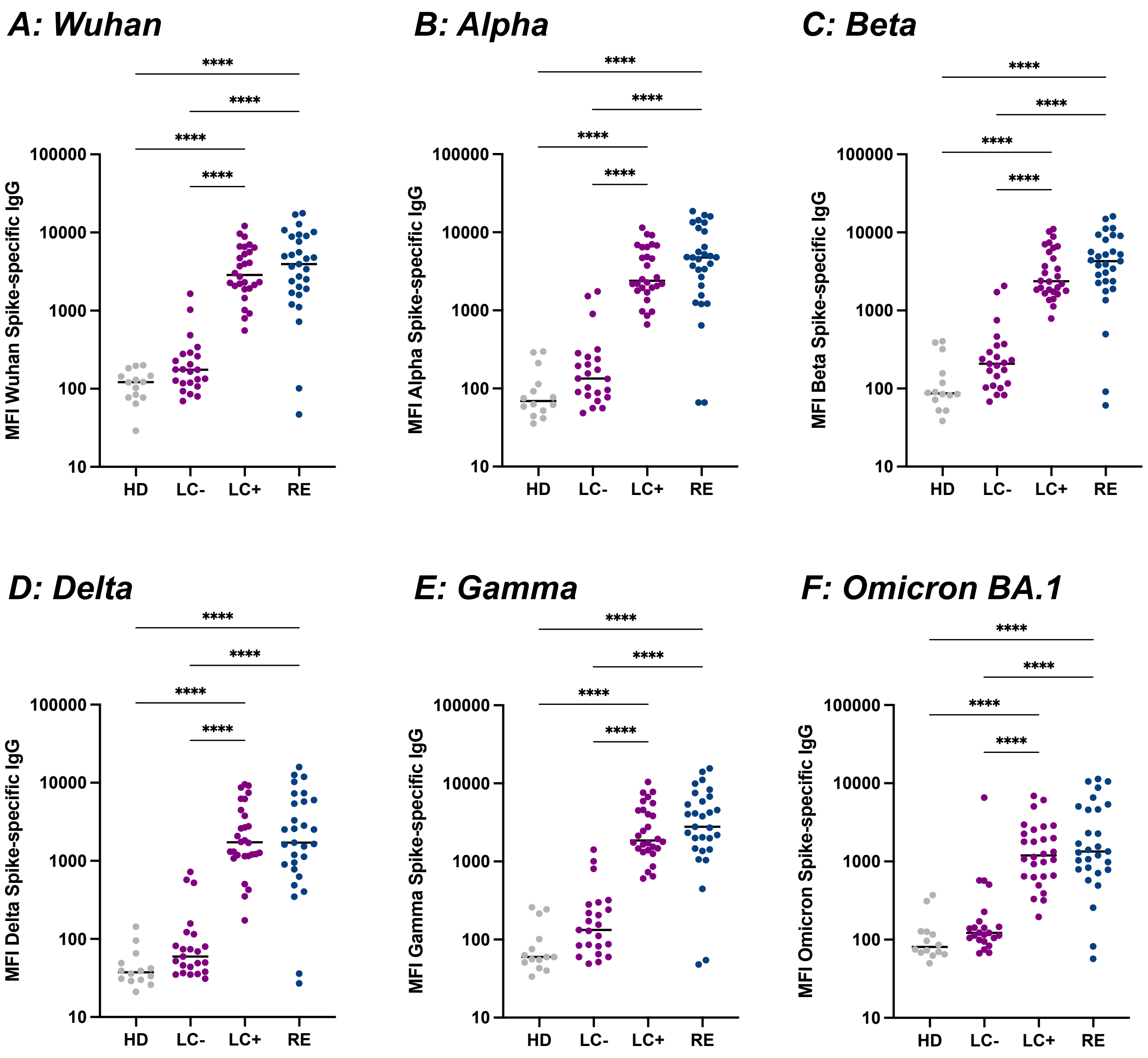

Supplementary Figure S1: antibody reactivity to the spikes of different SARS-CoV-2 variants

*(A-F) Antibodies specific to the spikes of the different SARS-CoV-2 variants of concern were measured in patient and control sera by a multiplexed Luminex assay. The spikes tested included that of the original Wuhan strain (A) and those of the Alpha, Beta, Delta, Gamma, and Omicron BA.1 variants (B-F), respectively. The mean fluorescent intensity (MFI) measured for human IgG is reported. HD: healthy donors; LC-: seronegative long COVID patients; LC+: seropositive long COVID patients; RE: recovered patients. Horizontal bars represent medians. Differences between groups were evaluated with the Kruskal-Wallis statistical test with Dunn's correction for multiple comparisons. **** P<0.0001.*

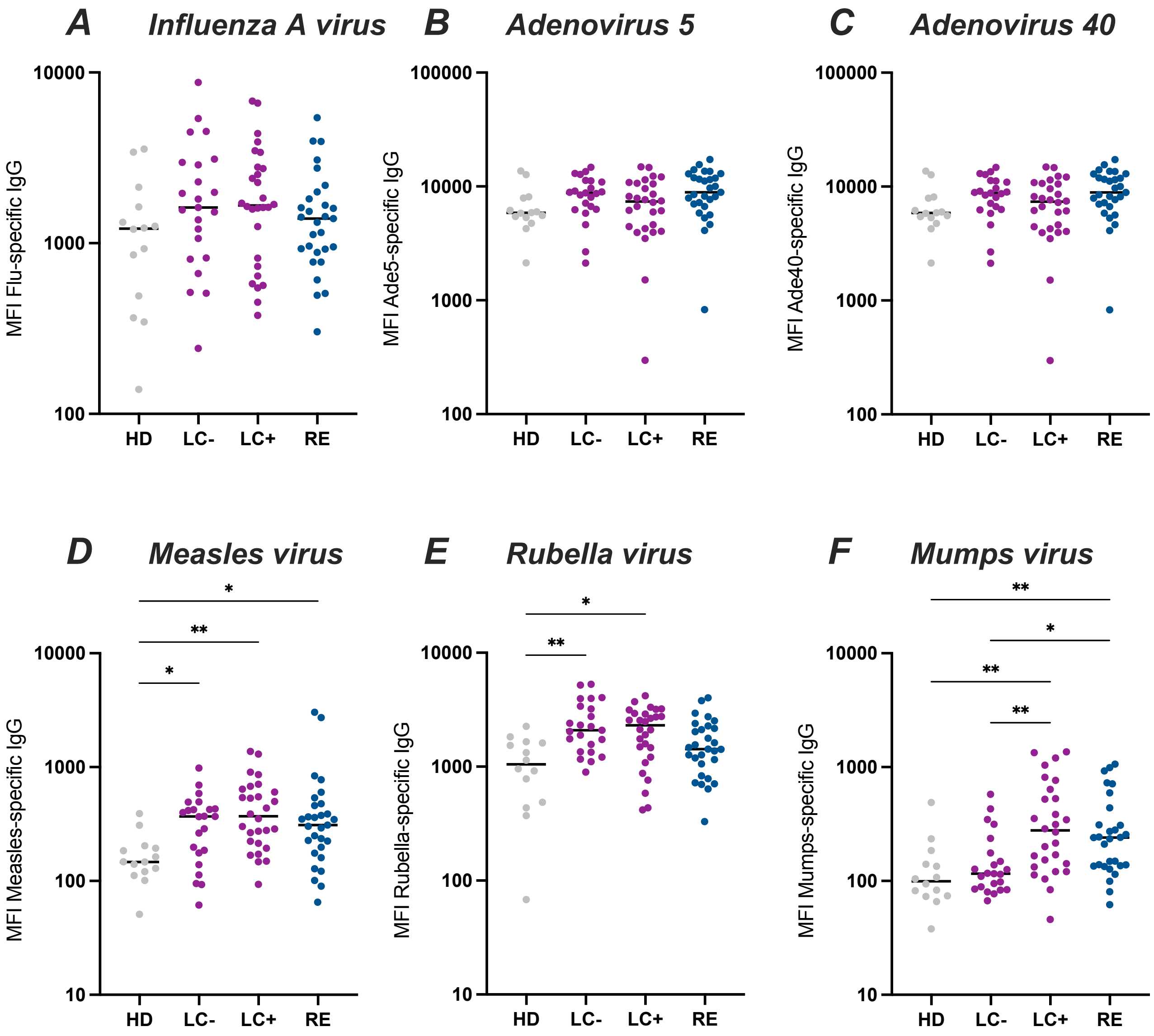

Supplementary Figure S2: antibody reactivity to other viruses

*(A-F) Antibodies specific to viruses unrelated to SARS-CoV-2 were measured in patient and control sera by a multiplexed Luminex assay. The antigens tested were derived from influenza A virus (A), adenovirus 5 (B), adenovirus 40 (C), measles virus (D), rubella virus (E), and mumps virus (F). The mean fluorescent intensity (MFI) measured for human IgG is reported. HD: healthy donors; LC-: seronegative long COVID patients; LC+: seropositive long COVID patients; RE: recovered patients. Horizontal bars represent medians. Differences between groups were evaluated with the Kruskal-Wallis statistical test with Dunn's correction for multiple comparisons. * P<0.05; ** P<0.01.*

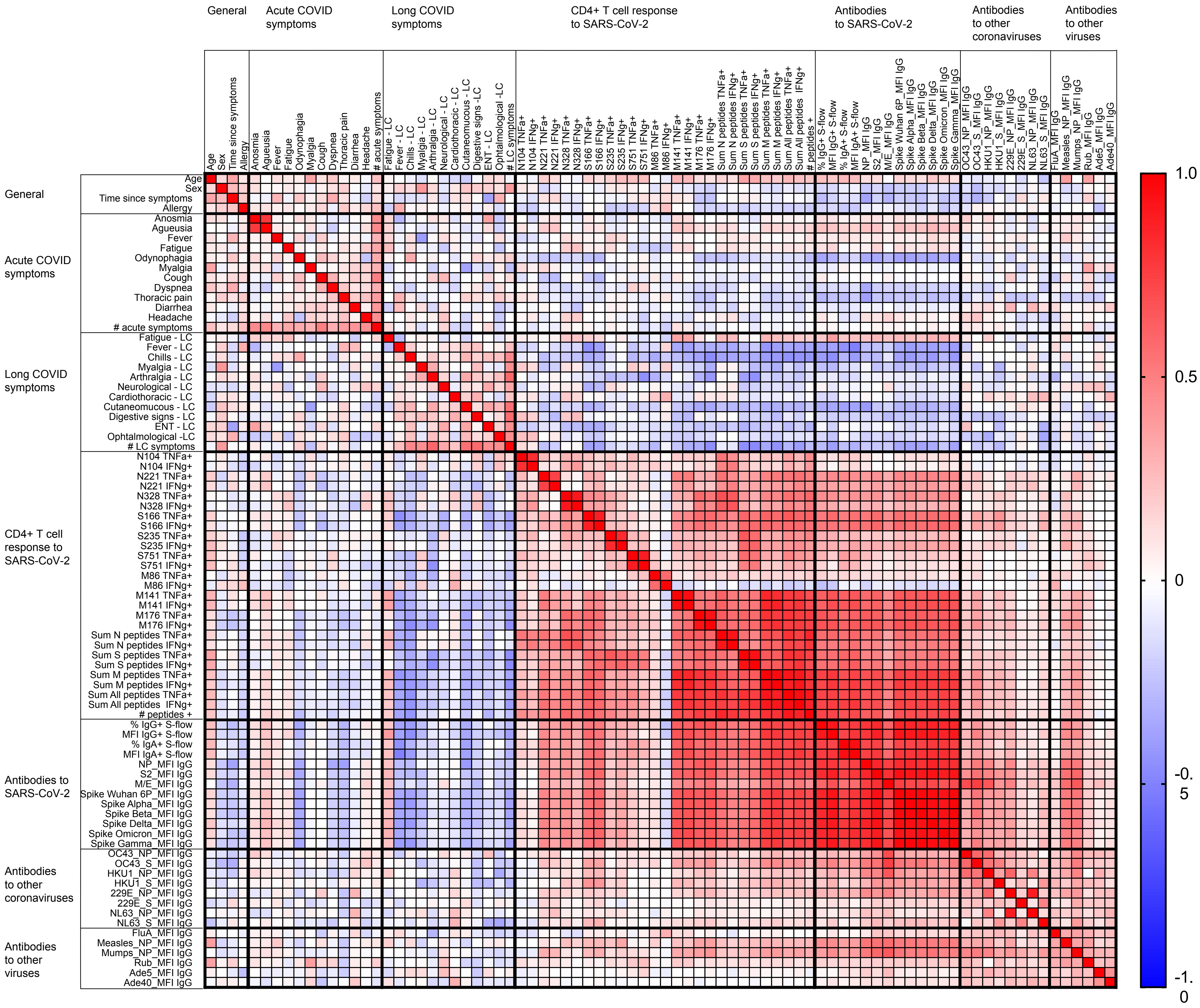

Supplementary Figure S3: correlation matrix of clinical and immunological parameters evaluated in the PERSICOT study

Spearman correlation coefficients are color-coded according to the scale bar (right)

Spike

S166	CTFEYVSQPFLMDLEGHQGN
S235	ITRFQTLLALHRSYLTPGDS
S751	NLLLQYGSFCTQLNRALTGI

Matrix

M86	CLVGLMWLSYFIASFRLFAR
M141	GAVILRGHLRIAGHHLGRCD
M176	LSYYKLGASQRVAGDSGFAA

Nucleocapsid

N104	LSPRWYFYYLGTGPEAGLPY
N221	LLLLDRLNQLESKMSGKGQQ
N328	GTWLTYTGAIKLDDKDPNKF

Supplementary Table 1: List of 20-mer peptides used to generate primary CD4+ T cell lines
Each peptide is named after the SARS-CoV-2 viral protein it originates from (S, M, or N) and the number of the first amino acid in the relevant protein.
